# Antibiotic Stewardship in the Management of Infected Diabetic Foot Ulcer Disease in Less Developed Countries

**DOI:** 10.1002/edm2.503

**Published:** 2024-06-25

**Authors:** Zulfiqarali G. Abbas, Raidah R. Gangji, Ilker Uçkay

**Affiliations:** ^1^ Muhimbili University of Health and Allied Sciences Dar es Salaam Tanzania; ^2^ Abbas Medical Center Dar es Salaam Tanzania; ^3^ Hubert Kairuki Memorial University Dar es Salaam Tanzania; ^4^ Infectiology Balgrist University Hospital Zurich Switzerland

**Keywords:** Africa, antibiotic stewardship, developing countries, diabetic foot infection, Gram stain, multi‐drug resistance, preemptive antibiotic therapy

## Abstract

**Background:**

Diabetic foot ulcers in developing countries often become infected. The healthcare systems are often not equipped to conduct the culture and the sensitivity tests required for prescribing a targeted antibiotic treatment for diabetic foot infection (DFI).

**Methods:**

We evaluate antibiotic stewardship programmes for DFIs, at every level of health care, with an emphasis on resource‐poor settings such as in Africa.

**Results:**

The management of DFI very often is adapted to the financial and practical realities of the resource‐poor regions. The application of the point‐of‐care Gram stain of deep tissue samples is efficient, rapid, low cost and ubiquitously available. Upon the identification of the predominant pathogen in the Gram stain, a semi‐quantitative preemptive antibiotic treatment can be started in accordance with the World Health Organization Aware, Watch and Restrict Essential Medicine List. This list is catered to every country and is a powerful tool. However, some basic knowledge of the local microbiological epidemiology is necessary to choose the most appropriate agent. We report our experience on using the rapidly available Gram stain for narrowing the preemptive choice of listed antibiotic agents, as an economic tool for antibiotic stewardship in DFIs.

**Conclusions:**

In the practical and resource‐saving management of DFI, the ‘therapeutic’ use of Gram stains is not common in resource‐rich countries but should be added to the arsenal of the general efforts for antibiotic stewardship.

## Graphical abstract

Diabetic foot infections (DFIs) in developing countries often are difficult to manage. The healthcare systems are often not equipped to conduct the culture and the sensitivity tests required for prescribing a targeted antibiotic treatment. We evaluate antibiotic stewardship programmes for DFIs, at every level of health care, with an emphasis on resource‐poor settings such as in Africa.

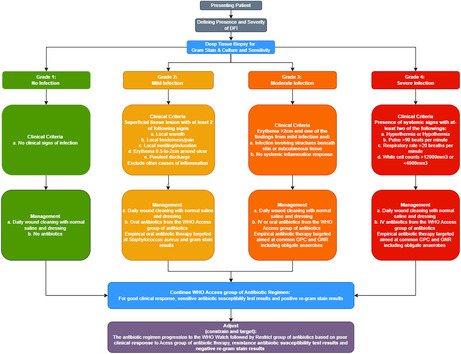

## 1 Introduction

Approximately 25% of adult patients with diabetics will develop a diabetic foot ulcer (DFU) during lifetime [1, 2]. Across the world, probably 40%–60% of all lower limb amputations are related to diabetes mellitus [1, 2, 3, 4, 5]. Diabetes mellitus is a major risk for up to one‐quarter of community‐acquired, or healthcare‐associated, orthopaedic infections: even in resource‐rich settings [6]. Diabetic foot problems might be more prevalent in persons with very limited access to professional health care, less medical and diabetes‐related education [7], malnutrition [8], lack of glycaemia control [7] or limited financial resources. At a certain level, the prevention, education and the approach to diabetic foot problems need a strong political backup, which is far beyond the preventive interaction [9] between the physician/surgeon and their patient.

In this article, we evaluate easily accessible tools and bedside methods for an efficient and resource‐saving (antibiotic) management of infections (diabetic foot infection [DFI]) in less developed setting, with an emphasis on Africa and issues of antibiotic over use, which is often due to a lack of diagnostic cultures. In contrast, we do not address the individual management of DFI in resource‐rich countries with performant insurance services and accessorily to innovative technologies, well‐equipped hospitals or a specialised health care [9].

## 2 Methods

For this narrative review, we performed a literature search in English and French languages regarding antibiotic stewardship in the management of DFI, both in developing countries and resource‐rich settings. We only included published scientific medical literature and emphasised on the usefulness of the Gram staining for the preemptive therapeutic use (not only for the diagnostic approach) community‐acquired DFIs. As there was sparse information published, we completed the review with our own experience and opinions.

## 3 Results

Many chronic DFU in developing countries become infected, which can be attributed to self‐surgeries, lack of compliance with follow‐up visits, herbal medication, living in (sub) tropical climates (less robust shoe ware), visiting faith healers, lack of insurances and microbiological laboratories and limited access to podiatrists, professional off‐loading, specialised surgery or angiology services; all of which contribute to the delayed presentation of DFI [1, 2]. Usually, the typical sequential timeline to seek medical help starts from home treatment, passing to a faith healer and/or herbalist, going to a primary healthcare centre and ultimately followed by a district or regional health centre. This odyssey may take up to 2 months. These delays to specialised (and relatively expensive) networks make DFIs very serious and costly [1, 2, 10, 11, 12, 13]. The positioning of antibiotic agents has a relatively greater role in the therapeutic arsenal when compared to resource‐rich settings that possess other therapeutic cornerstones of a successful approach to DFIs [1, 5, 10].

There exists sparse literature on the cost of managing infected DFU in developing countries. The bulk of the literature of these regions are based on microbiological surveys [10, 12]. A study determined the treatment costs from rural and urban regions in five different countries with various income economies [12]. The authors reported that the relative costs for managing DFI's would only be 1.6% of the comparative total costs in resource‐rich nations. This relative lower difference is also highly significant in other studies [1, 11, 13]. However, even if these costs are relatively low, they are very high in absolute numbers for the affected patients and their families. The cost of treating a chronic DFU in Tanzania equals 2–3 years of the average professional income [11]. In a single hospital in Trinidad and Tobago, the cost for the care of only 446 patients with DFI was $14 million per year [13], which the authors extrapolated to represent 0.4% of the entire domestic product. An antibiotic stewardship in developing countries should not only target resistances but should also optimise the sparse resources, by allowing a better availability of affordable antibiotic agents to a large number of patients in need of these agents [13, 14].

### 3.1 Antimicrobial Resistance in Diabetic Foot Infections in Resource‐Poor Countries

Frequently, in developing countries, DFI episodes are treated with (empirical) broad‐spectrum antibiotics for a prolonged duration and often without initial microbiological diagnosis of the causative pathogens [1, 14]. The lack of diagnostic microbiological cultures may lead to an over use of (not necessary) antibiotic agents, even if it is for a short period of time. New data from some resource‐poor settings suggest an increase in the proportion of multidrug‐resistant (MDR) Gram‐negatives in community‐acquired DFIs, of which the biggest burden would be due an increase in *Pseudomonas aeruginosa* [1, 5, 15, 16, 17, 18]. In settings with a high prevalence of (quinolone‐resistant) [18] *Pseudomonas* spp., clinicians might tend to cover *Pseudomonas*, and other nonfermenting rods, from the start with broad‐spectrum parenteral antibiotics (maybe even as a combination therapy) [19]. The environment of many specialised DFI centres, with regular infusions of broad‐spectrum agents, might be contaminated by MDR microorganisms, which has never been decontaminated to the best of our knowledge [20]. Even if many clinicians think that they can identify *P. aeruginosa* by its green colour, the macerated tissue, and by its characteristic grapefruit‐like smell, a recent prospective study (blinding physicians, wound care nurses and experienced surgeons) failed to correctly identify the presence, or absence, of *Pseudomonas* spp. by clinical judgement only [21].

Today, MDRs are daily reality for all clinicians, even in the poorest countries. Recent Turkish literature [17], and articles from the Southern Mediterranean [17, 22, 23], Asia [24] and (Sub)‐Saharan Africa [25, 26, 27, 28, 29, 30], indicate a high prevalence of *P. aeruginosa*, for which the reasons remain unknow. Several African studies from specialised diabetes centres and hospitals report a >60% resistance to third generation cephalosporins and an increasing resistance towards carbapenems among both, the Gram‐negative and Gram‐positive bacteria [23, 24, 25, 26, 27, 28, 29, 30], whereas in Asia, the microorganisms classified as MDR seem to shift towards more Gram‐negative bacteria [17]. Unsurprisingly, MDRs in DFI might contribute to a worse clinical prognosis when compared to their susceptible bacterial counterparts [17, 23, 24, 25, 26].

### 3.2 Rationale for a Therapeutic Approach Based on the Point‐of‐Care Gram Stains

In developing countries, the healthcare systems are usually divided into five levels [28]. The first two levels are primary services at district hospitals without facilities for bacterial cultures [1, 31, 32]. Even if a microbiological laboratory is available, it might be situated far away; so that many clinicians and patients might prefer to spend the money on antibiotic drugs rather than on the correct microbiological diagnoses or its transport costs. To overcome the limitation of laboratory and radiological facilities, multiple studies have reported using bedside tests to assess the microbiology of the DFI [1]. Among them, the utility of bedside Gram staining of the infected DFU allows the simple visualisation of invading bacteria, providing ground for a suitable (semi)‐preemptive, antibiotic treatment [33, 34, 35]. This is probably the easiest, most ubiquitous, and most standardised bedside microbiological bedside test available and surely the less expensive one.

The logic behind the Gram staining is threefold: Firstly, we presume that most prevalent microorganisms (and hence the most important causative pathogen groups for a given DFI episode) would equally be the most likely visible under the microscope. Secondly, we clinically target the most important bacteria group, letting less important co‐pathogens aside. Thirdly, the ‘stewardship power’ of the Gram staining prevails for the exclusion of a major Gram‐negative (and thus MDR) involvement. As Gram‐negative DFI pathogens are the bigger problem than Gram‐positives in terms of antibiotic stewardship, we are above interested not to see Gram‐negative pathogens in order to spare intravenous and broad‐spectrum antibiotic agents for complicated cases. Theoretically, the Gram staining could be used in two ways: It might replace microbiological cultures in very resource‐poor settings, especially for mild to moderate soft tissue DFIs that can be easily debrided. Or it can be used as a semi‐preemptive diagnostic solution while awaiting the culture results, by restringing the (unnecessary) empirical antibiotic choice for moderate‐to‐severe infections. The latter would also represent a valuable management option for resource‐rich countries.

However, and interestingly, there are not many publications available. The use of Gram staining for DFI is mentioned in some articles [18, 35], but its potential is usually not developed fully, with the exception of one publication stemming from the group of the first author of this review [33]. Abbas et al. reported that during the study period, they procured two deep tissue biopsy specimens using aseptic surgical technique and probing deep into the tissue at the base of the ulcer with sterile scalpel and forceps. The biopsies were placed in a container and taken immediately to the microbiology laboratory. All processing and isolate identifications were carried out by a trained laboratory technician [33]. The Abbas study revealed a relatively high positive predictive value of the Gram stain of deep tissue cultures with 86%–100%, for the growth of microorganisms with the corresponding Gram profiles. Among a total of 128 microbiological cultures, 118 (92%) yielded bacterial growth, 59 (50%) of these 118 cultures yielded mixed growth (80% included Gram‐negative organisms), and 38 (32%) and 20 (17%) yielded Gram‐negative and Gram‐positive organisms alone, respectively. Gram‐negative stains were 82% predictive of the growth of Gram‐negative organisms. There were just two discordant stain/culture pairs, with 96.4% congruency between the Gram stain appearance and ulterior microbiological culture results [33, 34, 35]. Similarly, the study conducted by Taniguchi et al. clearly demonstrated that Gram stains performed by healthcare professionals in the emergency department, directly contribute to the selection of significantly fewer broad‐spectrum antibiotics, reporting an estimate of 90% of the Gram stain‐based treatments were effective [35]. Moreover, the Gram stain may help in identifying the morphology of the bacteria, which aids in narrowing the possible common bacteria isolated in the infection site. In Tanzania, one Gram stain costs less than $1.0. In settings such as in Geneva, Switzerland, this is $US25 [1, 33, 36].

Besides many ‘Pros’, there might equally be ‘Cons’ regarding the Gram staining. The most obvious disadvantage of the Gram‐stain approach can be the missing of co‐pathogens in polymicrobial DFIs and the lack of detailed species classifications. For example, a Gram‐negative rod could be a susceptible *Proteus* spp., or an *Enterobacter* spp. naturally resistant to many first‐line β‐lactam antibiotics. The preemptive antibiotic treatment would be different, as a visual differentiation between these two species by Gram staining alone would require an advanced microbiological experience. On the other side, we are not obliged to treat all cultured (and all not culturable) pathogens in DFI, especially not in soft tissue infections. For example, many moderate and ischemic DFI harbour anaerobic co‐pathogens that we left to open air after debridement, and for which we regularly skip to administer a specific antianaerobic medication [37]. Practically, and according to international guidance [4, 5], we usually concentrate on the most one or two virulent pathogens, by interpreting additional pathogens either as contamination or as unimportant. This approach requires clinical experience. In that sense, the Gram stain cannot replace the value of a good clinical experience and decision‐making.

Likewise, the Gram stain shares another important shortcoming with the microbiological culture. In macerated and/or ischemic areas with polymicrobial, the Gram staining will not distinguish between contamination and causality of infection. Additionally, we need a minimal inoculum for an accurate Gram staining such as in an abscess. In paucibacillary mild infections and in infections that are spontaneously drained through the DFU, its performance might be reduced. Lastly, we ignore the interobserver variabilities in DFIs and the performance of the Gram staining under systemic antibiotic influence. In the literature, the Gram staining has been performed by experienced clinicians and/or microbiologists. We ignore its performance among less experienced clinicians or medical students, who are frequently involved in the DFI management in many resource‐poor countries. In contrast, we failed to detect major problems regarding technicality. The Gram‐stain approach can be applied in all levels of health care in developing countries.

### 3.3 Implementation of Other Stewardship Measures in Resource‐Poor Settings

The appropriate use of antibiotics for people living with diabetes, especially those with DFIs, calls for an antibiotic stewardship with personal commitment [6, 10, 38]. Besides the administration of narrow‐spectrum agents, one of the main cornerstones is less antibiotic use. The reduction in this use may occur in many ways [13, 38], whereas the presence of concomitant surgery and/or of iterative professional wound care certainly helps to achieve this goal. After surgery, most mild‐to‐moderate soft tissue infections can be treated for 1–2 weeks, and residual osteomyelitis, after resection of the bulk of bone infection, could be treated for 3–6 weeks [1, 5, 10, 14, 39]. Oral therapy can be applied from the start for chronic and moderate infections, especially if there has been surgery or debridement of all infected tissues [5, 39]. Antibiotic stewardship also can occur on a larger scope. For developing countries, especially the financing problems may become an important limitation factor (and, in turn, the financials outcomes of these programmes may dominate over the clinical outcomes). Ensuring development and access to regional [19] or international guidelines must be encouraged [4, 5]. The last Infectious Diseases Society of America (IDSA) clinical practice recommendations for the identification and treatment of DFI aim to reduce antibiotic resistance and the corresponding costs and adverse effects associated with antibiotic use [10]. Similarly, the World Health Organization 2019 has produced the AWaRe classification of antibiotics. The AWaRe classification, which is an acronym for ‘Access group, Watch group and Restrict group’ of antibiotics was first introduced in 2017 for the Essential Medicine List (EML)‐listed antibiotics (https://extranet.who.int/ncdccs/Data/ken_D1_clinical%20guidelines%20for%20management%20and%20referral%20of%20common%20conditions.pdf). The EML List is available in every country. However, the EML of developing countries are limited to 20–30 antibiotic choices [40].

## 4 Discussion

**FIGURE 1:**
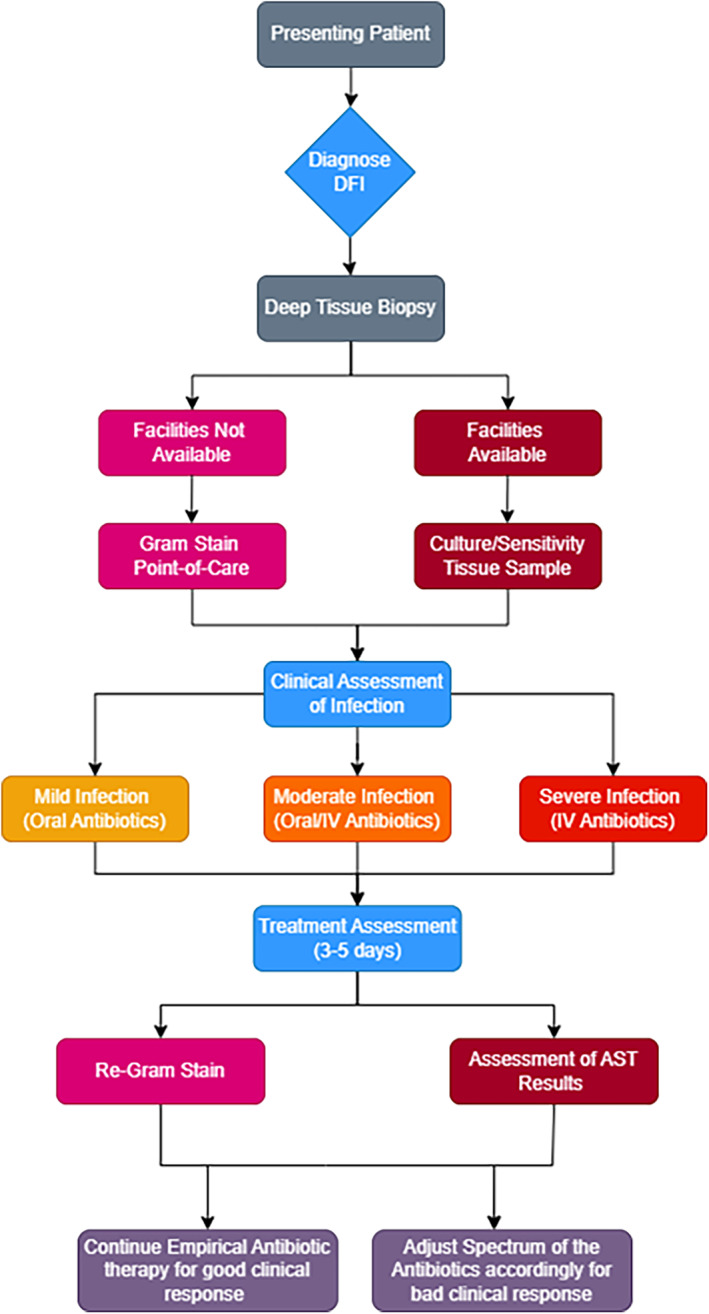
Flow chart showing the cascade of implementing antibiotic stewardship. In areas where culture and sensitivity facilities are not available an alternative of point‐of‐care Gram stain can be utilised to prescribe antibiotics appropriately and reducing the emergence of MDR bacteria.

**FIGURE 2:**
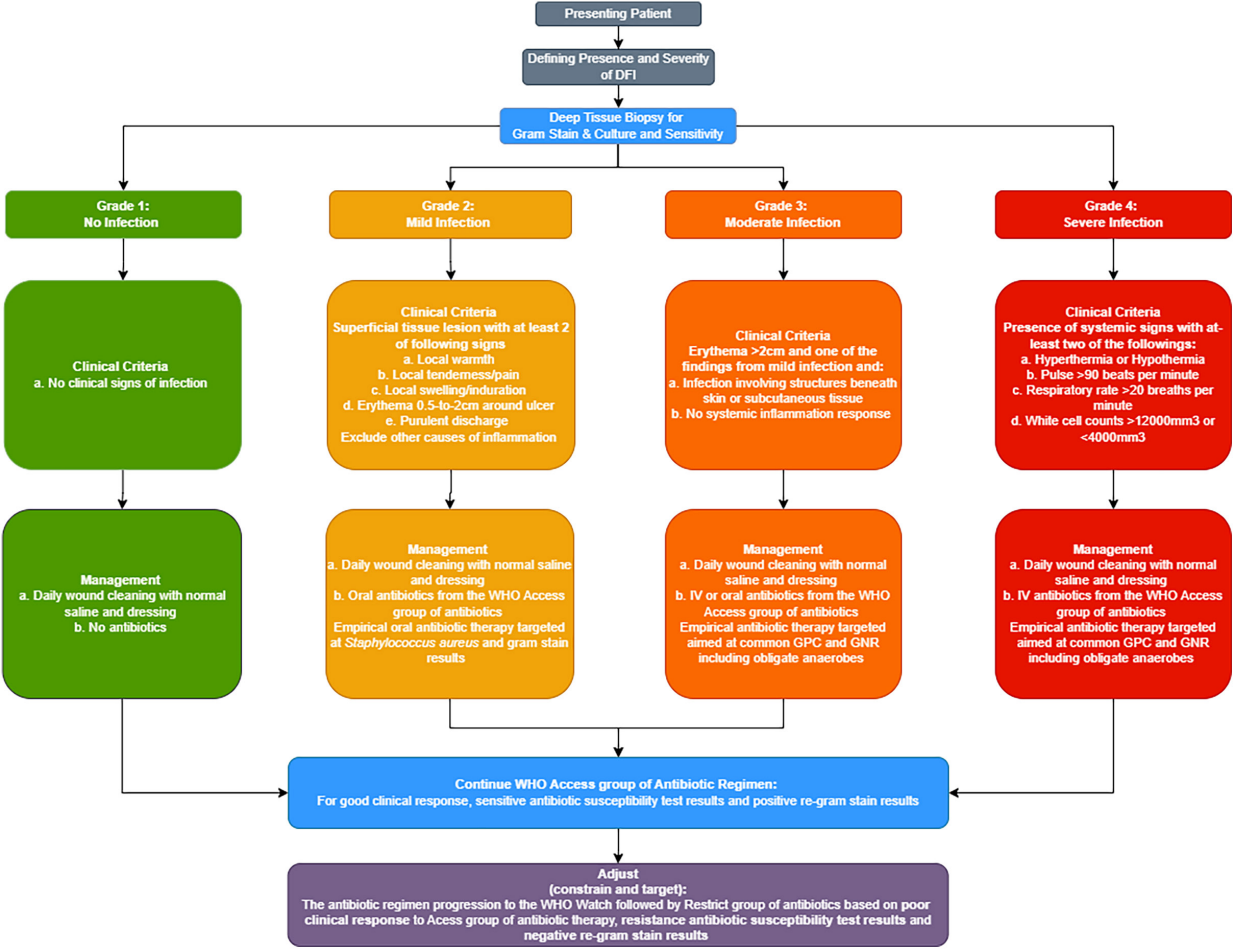
Algorithm for implementing antibiotic stewardship from the WHO, AWaRe and International Working Group of the Diabetic Foot (IWGDF) for developing countries.

The lack of bacterial culture facilities in developing countries limits the option of antibiotic stewardship. Therefore, after reviewing the current antimicrobial stewardship implementation programmes available, we have integrated and combined an antimicrobial stewardship algorithm for community‐acquired DFIs in Dar es Salaam that can be implemented in resource‐poor countries, based on the Gram staining. The flow chart below (Figure 1) gives a brief on the cascade that would follow in an area with limited culture and sensitivity facilities. The presenting patient (Figure 2) with DFI should be diagnosed according to the International Working Group of the Diabetic Foot (IWGDF) guidelines. Deep tissue biopsy should be taken where facilities for culture are available tissue culture should be conducted but if facilities for culture are not available point‐of‐care Gram stain should be done. Antibiotic treatment protocols based on Gram stains in places that lack the resources for definite diagnosis is a noble approach, which can be used easily in many resource‐poor countries. The main limitation of our work is the paucity of the available literature to be reviewed and the unusual format of the manuscript, which is a mix of review and expert opinion paper. However, we also think that the message is important, and hopefully an impetus for research.

### 4.1 Conclusions

Antibiotic stewardship in necessary for the management of DFI cases, equally in developing countries. Unnecessarily prescribing antibiotics for noninfected diabetic feet and over‐prescribing antibiotics for mild or moderate DFIs very presumably contribute to the development of MDR bacteria. In moderate DFIs, we do encourage clinicians to gain more experience by using the easy, rapid and cost‐effective Gram staining in many settings, including the resource‐poor and the resource‐rich settings, and to tailor a preemptive therapy accordingly instead of prescribing a global large‐spectrum treatment. We equally need further research regarding cost reduction and associated outcomes with the therapeutic use of the Gram stain in both, the developing and the rich countries.

## Author Contributions

**Zulfiqarali G. Abbas:** conceptualization (lead), project administration (supporting), resources (supporting). **Raidah R. Gangji:** methodology (supporting), resources (supporting), writing–original draft (supporting). **Ilker Uçkay:** conceptualization (supporting), project administration (lead), resources (lead), supervision (lead), writing–original draft (supporting), writing–review and editing (lead).

## Consent

The authors have nothing to report.

## Conflicts of Interest

Z.G.A. and I.U. are active members of the Working Group ‘Infections’ at the 2023 International Working Group of the Diabetic Foot (IWGDF). Z.G.A. is the current President of D‐Foot International.

## Data Availability

The authors have nothing to report.
